# Are average years of education losing predictive power for economic growth? An alternative measure through structural equations modeling

**DOI:** 10.1371/journal.pone.0213651

**Published:** 2019-03-21

**Authors:** Henry Laverde-Rojas, Juan C. Correa, Klaus Jaffe, Mario I. Caicedo

**Affiliations:** 1 Facultad de Economía/Universidad Santo Tomás, Bogotá, Colombia; 2 Facultad de Psicología/Fundación Universitaria Konrad Lorenz, Bogotá, Colombia; 3 Departamento de Biología de los Organismos/Universidad Simón Bolívar, Caracas, Venezuela; 4 Departamento de Física/Universidad Simón Bolívar, Caracas, Venezuela; University of Zilina, SLOVAKIA

## Abstract

The accumulation of knowledge required to produce economic value is a process that often relates to nations economic growth. Some decades ago many authors, in the absence of other available indicators, used to rely on certain measures of human capital such as years of schooling, enrollment rates, or literacy. In this paper, we show that the predictive power of years of education as a proxy for human capital started to dwindle in 1990 when the schooling of nations began to be homogenized. We developed a structural equation model that estimates a metric of human capital that is less sensitive than average years of education and remains as a significant predictor of economic growth when tested with both cross-section data and panel data.

## Introduction

Substantial evidence shows that human capital plays a critical role in nations economic growth [1, 2]. Since its initial conception [3, 4], human capital is said to capture the stock of knowledge and cognitive abilities required to produce economic value. Many authors regard human capital as a result of schooling, and therefore employ educational variables such as average years of education (*AYE*) as a proxy indicator [5, 6]. Some scholars have criticized this latter metric for several reasons: *i*) it omits the quality of education, *ii*) it assumes homogeneity among individuals, *iii*) it is insensitive to educational systems, *iv*) it ignores human capital from unschooled people and *v*) it only evaluates a single component of a broader concept [7–9].

Given the limitations of *AYE*, some researchers use indicators of educational quality [5, 6] or health [10] among others variables [11]. Other scholars have suggested the estimation of human capital in a way most elaborate using nonparametric and parametric techniques (for a detailed discussion see [8]). A usual method of the latter technique is Principal Component Analysis (PCA). As a dimensional reduction technique, PCA lacks to analyze different aspects of the nature of human capital. Human capital cannot be conceived as a one-dimensional construct but instead as the composite resulting from several variables. Furthermore, as human capital influences workers productivity, their expected returns and their ability to create and absorb new productive technologies, this capital posses the double connotation of being input and outcome of different variables. Consequently, estimates of human capital should reflect its abstract, multidimensional and directional nature.

**Fig 1:**
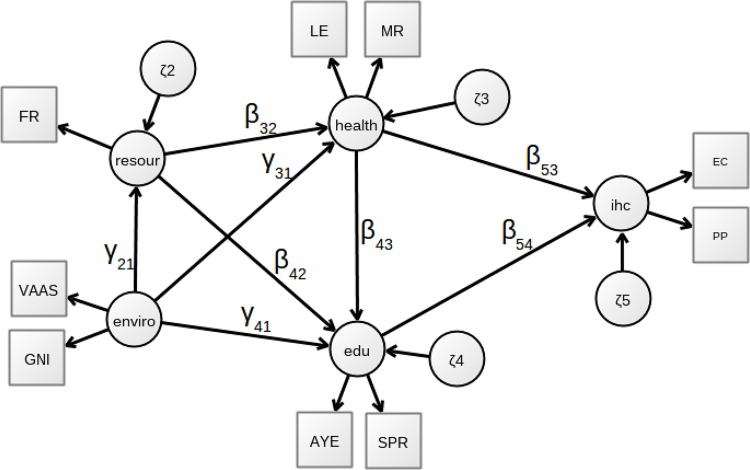
Path diagram—PLS-PM of human capital. Circles are latent variables, and boxes are observable variables. The arrows represent dependence relationships between the variables.

Messinis and Ahmed [12] have approximated human capital through PCA. In contradistinction, we adopt a structural equation modeling framework [13], being this a technique widely and successfully used by psychologists and sociologists though it is not frequently used by economists focused on a macroeconomic approach to human capital. Structural equation modeling is a multivariate statistical analysis method which combines factor analysis and multiple regression, and it is employed to analyze the relationship between measured (observable) variables and latent constructs (non-observable variables) [14]. Following this approach, we posit a system of equations where direct and indirect effects can be estimated to disentangle the relationships that exist between the process of accumulating human capital (i.e., education, health, household backgrounds, etc.) and their returns and outcomes (e.g., productivity increase, generation of new knowledge, etc.). This system of equations is entirely equivalent to the path diagram shown in Fig 1.

Our approach allows us to present two contributions. Firstly, it enables us to tackle the problems of omitted variables commonly known in econometrics [15] while explicitly specifying the relationships between the variables that influence the determination of human capital in a more comprehensive way. Secondly, and not least significant, it extends the results of Messinis and Ahmed [12] by testing the robustness of our index of human capital in cross-section and panel data, and testing the hypothesis that the significant positive effect of schooling could only be observable once a country crosses a determinate threshold, circa ten years of education per capita [16]. Our results support the critics on the use of AYE as a proxy for human capital; nevertheless, it is fair to recall that AYE, literacy and school enrollment are indices available for every country, and in fact, decades ago they were the only available data.

So far, the proposal of an index of human capital that connects its inputs with its returns, while considering the direct impact of available resources and socio-economic conditions is missing in the literature. Our goal in this paper is to give a step in the direction of filling this gap.

## Materials and methods

### Methodology

As an abstract concept, human capital reflects the abilities and cognitive skills resulting from an accumulation of factors such as education, health, innate talents, etc., that form a potential stock. These factors allow individuals to generate a series of returns like productivity, innovation and inventiveness capacity. These returns are affected by the quality of the inputs used in the accumulation of potential human capital. For example, the quality of individuals’ education and health depends on the resources allocated by households to these areas and on countries socio-economic conditions, among other elements. As stated in the introduction, we used a structural equation model (SEM) to preserve the notions of human capital mentioned above. This SEM approach consists of a structural part, which relates latent variables, and a measurement part that associates observable and latent variables, simultaneously estimated, as shown in the path diagram of Fig 1. For the sake of completeness, we show the structural model obtained from the path diagram through the following equation system

$$resour=\gamma_{11}enviro+\zeta_{1}$$ (1)

$$health=\beta_{21}resour+\gamma_{21}enviro+\zeta_{2}$$ (2)

$$edu=\beta_{31}resour+\beta_{32}health+\gamma_{31}enviro+\zeta_{3}$$ (3)

$$ihc=\beta_{42}health+\beta_{43}edu+\zeta_{4}$$ (4)

The meaning of the parameters *γ*_*ij*_, *β*_*ij*_, and *ζ*_*i*_, *i* = 1, 2, 3, 4 in the equations is clear, the *γ*_*ij*_’s and *β*_*ij*_’s show the linear relationships between both the exogenous and endogenous latent variables. The observable variables contribute to the latent ones, and the contribution comes to form an extra set of equations which, for reasons of readability, we do not show here. Instead, we refer the interested reader to [14] for technical details. Finally, the *ζ*_*i*_’s represent errors or residual terms. The estimation of all these parameters employs a partial least squares path modeling (PLS-PM) technique [17]. The estimates from this technique adjust more appropriately to the concept of human capital and are widely robust to numerous weaknesses such as bias indicators, multicollinearity or missing specification of the structural model, and the scores of latent variables are closer to the true values [18].

The complete model is estimated using standardized data, on reflective mode, with a centroid scheme (for technical details, see [17]). As PLS-PM does not rely on distributional assumptions, a bootstrapping process is regularly used to assess the fit of the structural model [19]. Furthermore, the validity and reliability analysis of the measurement model is carried out to evaluate whether or not the theoretical concepts are measured correctly by the observed variables (for details see the online appendix at: https://arxiv.org/abs/1807.07051).

Having given a rough description of our model, we now turn to a detailed description of the variables we used. We begin by stating that we looked for several sets of observable variables (observable in the sense that they are available in public databases), one of us (HL) performed a sensitivity analysis and concluded that the variables that we ended up with provided us with a minimal set of independent observables.

In our model, *enviro* is the only exogenous latent variable encompassing the socio-economic environment. As a latent variable, *enviro* is built from two observables, namely the value-added contributed by the agricultural sector to GDP (VAAS), and a binary variable that classifies countries according to their respective gross national incomes (GNI) per capita during the study period. This latter variable is based on the classification of the World Bank that sorts economies in ascending order into low-income, middle-income (which is further subdivided into lower-middle and upper-middle) and high-income groups based on GNI per capita. To simplify its estimation and interpretation, we decided to synthesize it into a binary variable: 1 for upper-middle and high incomes and 0 otherwise. The economic development of a country is a fundamental element for the expansion and use of human capital. For developing countries, these conditions are linked to the prosperity of a particular sector, such as agriculture. Nevertheless, there are various positions regarding the relationship between the value added of agriculture and economic development. In general, all these positions consider a reallocation of resources from the agricultural sector to the industrial sector to be a condition of development [20, 21].

Our next variable, *resour*, *η*_1_, is the first endogenous latent variable. *resour* is an attempt to model household resources. We base our latent concept on the economic idea that household size is an essential factor in the formation of human capital. Indeed, small families can assign large amounts of attention and resources to their children while large families can allocate little resources to each child. To summarize this idea, we consider a contribution from high fertility rates (FR) which clearly constrain the formation of human capital [22, 23].

It is clear that *health*, *η*_2_, is our second endogenous latent variable. *health* has a direct effect on both education and our index of human capital. We propose to use life expectancy (LE) and the mortality rate for children under five years (MR) as observable variables that contribute to measuring health.

The next latent concept we address is education, as described by the endogenous variable *edu*, *η*_3_, which as usual gets its main contribution from the observable average years of education (AYE). We propose to consider a contribution from another observable, the student-professor ratio (SPR) which we think might correct, to some extent, for the quality of education.

Our last latent variable *ihc*, *η*_4_, stands for returns on human capital. Two observables are considered to contribute to *ihc*. To introduce them we follow a natural assumption, that individuals’ cognitive abilities have a direct impact on their productivity and innovative capacity. Patent applications by residents per capita (PP) look like a good measure of creative capacity, while energy consumption per capita (EC) seems fit as a measure of productivity. EC is also used to prevent problems of circularity (namely; we use measures related to GDP to measure the indicator and then return it with the GDP growth rate) in later empirical applications of economic growth.

Given the variables above and the path diagram shown in Fig 1 which is equivalent to the system of equations, we can quickly notice that the returns of human capital (*ihc*) have two major interrelated components: education (*edu*) and *health*, and *health* also affects educational attainment [24] but is in turn influenced by *resour* and *enviro*.

In this way, we see that the quantity and quality of human capital depend on the resources devoted by households (*resour*) as well as on the background and the socioeconomic context of the countries (*enviro*) [5]. Finally, we note that the socioeconomic environment has a direct impact on the household resources.

Once the composite indicator is built, we seek to test it vis-à-vis with AYE in economic growth models. Our cross-country analytic framework is as follows:

$$\vartheta_{i}=\beta ihc_{i}+X_{i}^{{}^{\prime }}\theta +\mu_{i}+\epsilon_{2i}$$ (5)

where *ϑ*_*i*_ is the average growth rate of GDP per capita of country *i* in the observed period; *ihc* is the index of human capital for country *i*; *X*_*i*_ is a vector of control variables; *μ*_*i*_ is a specific component that captures the existence of other determinants of growth not included in *X*_*i*_ for each country, these components are not observable. The sign and significance of *β* are the target of interest. Estimating Eq 5 presents some problems. A first drawback to consider is the treatment that should be given to the specific component. Estimating 5 will be valid only if the individual component can be considered as uncorrelated with the other explanatory variables, which is analogous to the problem of omitted variable bias. Another difficulty arises from the endogenous response of some variables in 5 to changes in GDP, particularly *ihc*. We used an instrumental variable approach to deal with these problems. While Eq 5 is the second stage, the first stage will be represented by:

$$ihc_{i}=X_{i}^{{}^{\prime }}\psi +Z_{i}^{{}^{\prime }}\lambda +\epsilon_{1i}$$ (6)

where *Z*_*i*_ is a vector of instruments.

There has been a growing concern about the strength and validity of the instrumental variables in practice. Obtaining instruments is a complex task [25], where much of the literature that builds “smart” instruments might be invalid, *E*[*Z*_*i*_*ϵ*_1*i*_] ≠ 0, or weak, *E*[*Z*_*i*_*ihc*_*i*_] ≠ 0 but with low correlation, or both [26]. Using lagged values and initial values is a common practice in the literature of economic growth. However, the use of these instruments may be an imperfect way to treat the problem, particularly if these variables show specific components or if global trends may alter significantly over time. New developments in econometrics have assisted in the search for a better identification, particularly in the context of panel data with the emergence of the System GMM estimator from [27] and [28]. In cross-section data, Lewbel [29] introduced a new method for identifying structural parameters in models where instrumental variables are neither available nor valid or weak. The identification arises from having regressors not correlated with the product of heteroskedasticity errors. Specifically Lewbel [29] showed that the identification of the parameters in Eq 5 is possible if

$$E\left[X_{i}\epsilon_{1i}\right]=0,\;E\left[X_{i}\epsilon_{2i}\right]=0,\;cov\left[Z_{i},\epsilon_{1i}\epsilon_{2i}\right]=0,\;cov\left[Z_{i},\epsilon_{2i}^{2}\right]\neq 0$$ (7)

where *ϵ*_1*i*_ and *ϵ*_2*i*_ are errors of the first and second stage, respectively; and *Z*_*i*_ is a vector of exogenous variables, which can be a subset of *X*_*i*_ or *Z*_*i*_ = *X*_*i*_. The implementation is carried out by regressing each endogenous variable with all exogenous variables and recovering the residuals vector $\hat{\epsilon }$. Then, these residuals are used to create instruments by means of the product $\left[Z_{i}-\bar{Z}\right]\left(\hat{\epsilon_{2}}\right)$, where $\bar{Z}$ is the mean of *Z*_*i*_. As Lewbel [29] notes, the assumption that *cov*[*Z*_*i*_, *ϵ*_1*i*_*ϵ*_2*i*_] = 0 means $\left[Z_{i}-\bar{Z}\right]\left(\hat{\epsilon_{2}}\right)$ is a valid instrument because it is uncorrelated to *ϵ*_1*i*_. The force of the instruments will then be proportional to the degree of heteroskedasticity of *ϵ*_2*i*_ with regard to *Z*_*i*_. Thus, identification requires that the error terms of the regression of the first stage are heteroskedastic. As Lewbel [29] mentions, this assumption can be verified by a Breusch-Pagan test. Although the estimate can be made by 2SLS, in the presence of heteroskedasticity, efficiency can be increased by GMM [30]. Following Lewbel’s approach, we let *S* to be a vector of elements *ϑ*_*i*_, *ihc*_*i*_, *X*_*i*_ and *Z*_*i*_ and define Ω as the set of parameters of the reduced form of 5 and 6, then

$$g_{1}\left(S,\Omega \right)=X\left(\vartheta_{i}-\alpha +\beta ihc_{i}+X_{i}^{{}^{\prime }}\theta \right)$$

$$g_{2}\left(S,\Omega \right)=X\left(ihc_{i}-\sigma +X_{i}^{{}^{\prime }}\psi +Z_{i}^{{}^{\prime }}\lambda \right)$$

$$g_{3}\left(S,\Omega \right)=Z_{i}-\bar{Z}$$

$$g_{4}\left(S,\Omega \right)=\left(Z_{i}-\bar{Z}\right)\left(\vartheta_{i}-\alpha +\beta ihc_{i}+X_{i}^{{}^{\prime }}\theta \right)\left(ihc_{i}-\sigma +X_{i}^{{}^{\prime }}\psi +Z_{i}^{{}^{\prime }}\lambda \right)$$

Pooling these vectors in a large vector *G*(*S*, Ω) and satisfying the orthogonality conditions in Eq 7, *G*(*S*, Ω) = 0 must be met, allowing for correct estimation of the structural parameters of 5 by GMM.

To further evaluate the robustness of our index, we proceed with a panel data analysis. Following the reasoning of cross-section data, Eq 5 can be set to panel data models as follows:

$$\Delta y_{i,t}=\left(\alpha -1\right)y_{i,t-1}+x_{i,t}^{{}^{\prime }}\beta +\mu_{i}+\delta_{t}+v_{i,t};\;i=1,\ldots ,N;\;t=2,\ldots ,T$$ (8)

$$E\left(\mu_{i}\right)=E\left(v_{i,t}\right)=E\left(\mu_{i}v_{i,t}\right)=0$$

where Δ*y*_*i*,*t*_ is the log difference of GDP per worker; *y*_*i*,*t*−1_ is the GDP per worker in the first year of the period; $x_{i,t}^{{}^{\prime }}$ is a vector of variables of countries’ own characteristics (including human capital); *μ*_*i*_ are unobserved country-specific effects and *δ*_*t*_ includes temporary effects affecting different countries. To address these temporary effects a set of time dummies for each regressions is included. Eq 8 is equivalent to the estimation of a model of dynamic panels with lagged dependent variable on the right side

$$y_{i,t}=\alpha y_{i,t-1}+x_{i,t}^{{}^{\prime }}\beta +\mu_{i}+\delta_{t}+v_{i,t};\;i=1,\ldots ,N;\;t=2,\ldots ,T$$ (9)

The estimation of Eq 9 faces some problems for identification. One of them is unobserved country-specific effects, *μ*_*i*_ (e.g., the initial value of unobserved technology, preferences or those relating to socio-economic environment) which could be correlated with other regressors leading to biased parameter estimations. Models of panel data avoid this problem by treating those individual characteristics as time-invariant and eliminating them through transformations. Another problem is the presence of endogeneity in some variables in $x_{i,t}^{{}^{\prime }}$. As usual, one can resort to the instrumental variables approach. However, the difficulty is to find valid and strong instruments. Finally, the value of the lagged dependent variable, *y*_*i*,*t*−1_, is correlated with fixed effects on the error term.

A standard approach in the literature to deal with these problems is to use the difference GMM estimator developed by Arellano and Bond [31]. This estimator transforms (first difference) the variables to eliminate fixed effects and, subsequently, endogenous variables are instrumented (including the predetermined variables) with the lags of the variables in levels. However, the estimator of Arellano and Bond [31] suffers from sampling bias when the number of periods is small and the dependent variable shows a high degree of persistence [32]. Bond et al. [33] recommend the system GMM estimator developed by Arellano and Bover [31] and Blundell and Bond [28] to get more consistent estimates instead. The system GMM estimator uses lagged values in levels (dated on t-2 or earlier) as instruments for the transformed variables in the equation of initial differences, as does Arellano and Bond [31], but added lagged differences are instrumental to the endogenous variables in the levels equation. By combining these two equations is possible to improve the efficiency of estimates and to avoid sampling bias. However, the gain of the asymptotic efficiency comes at a cost. The number of instruments tends to increase exponentially with the number of periods [34]. This proliferation of instruments can lead to different sources of bias, such as large estimated variance matrix, downward bias in the standard errors in the two stages of the estimation, weakened over-identification test, and overfitting of the endogenous variables. A golden rule regarding estimates is that the number of instruments does not exceed the number of groups. Following Roodman’s notes [34] this study uses the System GMM estimator with the second lag for both differences in levels equation. The consistency of the estimates relies on compliance with the orthogonality conditions (i.e. that residuals are not serially intercorrelated and regressors are exogenous). The examination of these assumptions supposes the use of the Hansen J tests to check the validity of the instruments, and the AR(2) test to discarding serial correlation.

### Data

The data covers the period between 1970 and 2011 sampling 91 countries with different development levels. We introduced all variables as the averages of the said period. We took GDP per capita from Penn World Table (PWT), version 8.0 [35]. As we already described our metric *ihc*, we compared our results for *ihc* with average years of education, as developed by Barro and Lee [36] as well as the pupil–teacher ratio in elementary school taken from World Bank indicators.

To implement Lewbel’s approach, we selected the control variables following common practice from specialized literature on economic growth [37]. The variables included are divided into two groups. The first one includes the investment in physical capital, measured as the average share of investment real to GDP, average government consumption as a percentage of GDP, both variables taken from PWT, and inflation measured by consumer prices from the World Bank indicators. The second group includes population growth rate, taken from PWT, a binary variable measuring the level of democracy in the countries and two estimated indicators by principal component analysis to approximate the degree of impugnment of the countries, these three last variables come from the [38] database. Given the endogeneity problems of human capital, investment and population growth rate were instrumented with their initial values in 1975 and their lags in 1970. The data is avalaible at: https://dataverse.harvard.edu/dataset.xhtml?persistentId=doi:10.7910/DVN/WF37MN.

## Results

In the following tables, we present the non-standardized coefficients. However, to compare the influence of both *ihc* and *AYE* we interpret the results upon standardized coefficients. Table 1 shows the results of the regressions without controlling the problems previously mentioned (e.g., endogeneity, omitted variables, etc.).

**Table 1:** Regression analysis for economic growth by OLS.

	Dependent variable: log difference GDP							
	(1)	(2)	(3)	(4)	(5)	(6)	(7)	(8)
Constant	0.068 (0.432)	-0.502 (0.606)	6.466*** (1.601)	2.013 (1.398)	4.836* (2.729)	0.523 (2.408)	6.989** (2.823)	5.739** (2.584)
log(*ihc*)	0.321*** (0.066)		0.774*** (0.113)		0.659*** (0.136)		0.540*** (0.172)	
log(*AYE*)		1.325*** (0.308)		2.070*** (0.524)		1.696*** (0.535)		1.297*** (0.477)
log(GDP75)			-1.069*** (0.245)	-0.460** (0.225)	-1.161*** (0.238)	-0.701*** (0.239)	-1.205*** (0.278)	-0.914*** (0.252)
N	91	91	91	91	91	91	91	91
*R*^2^	0.201	0.176	0.372	0.226	0.445	0.353	0.489	0.466

Note: Huber-White standard errors. All variables are averages for the period 1975-2011. The table presents non-standardized coefficients Statistical significance:

*p<0.1,

**p<0.05,

***p<0.01.

Where: *ihc*, the index of human capital; *AYE*, average years of education; GDP75, initial value of GDP. Controls used: government share as a percentage of GDP, inflation, investment in physical capital to GDP, population growth rate, level of democracy, contestation, inclusiveness and a dummy for African countries.

Column (1) shows a simple regression between the logarithm of *ihc* and the growth rate of GDP. The coefficient of *ihc* suggests that human capital impacts economic growth positively and significantly, with an explained variance of 0.201. In column (2) these results are contrasted with the traditional *AYE*, which also contributes significantly to economic growth with an R-squared of 0.176. In columns (3) and (4) the initial value of GDP is added to assess conditional convergence across countries. By including this variable in the regression, the relationship remains highly significant to *ihc* (column 3) increasing its explained variance to 0.372. The results for *AYE* (column 4), although highly significant, are of lesser magnitude with an *R*^2^ = 0.226. Columns (5) and (6) include government consumption as a percentage of GDP, inflation and investment in physical capital as control variables. Although the introduction of these variables marginally decreases the magnitude of *ihc*, it retains its statistical significance. In this new specification, *AYE* remains highly significant, but it has less impact compared with *ihc* (a difference of 0.575, with increases in standard deviations) and contributes in a lesser extent on the explained variance. Columns (7) and (8) introduce five additional control variables: population growth rate, a binary variable that measures the level of democracy in the countries, two indicators that approximate the extent of impugnment of countries and a regional variable for African countries. These variables do not significantly alter the performance of *ihc*, which always exhibits higher levels of impact and explained variance than *AYE*.

These results support the performance of *ihc* as a determinant of economic growth, but they could be spurious because of endogeneity [1]. To overcome this problem, the use of instrumental variables is advisable [25] [26] [28]. We evaluated the validity of the instruments through the Hansen J statistic, which allows us to verify that orthogonality conditions are met. Furthermore, we evaluated the problem of weak instruments [39] using a model based on the Kleibergen-Paap rk Wald F statistic which is robust in the presence of heteroskedasticity [40]. Table 2 shows the estimates by the Generalized Method of Moments (GMM).

**Table 2:** Regression analysis for economic growth by GMM.

	Dependent variable: log difference GDP							
	(1)	(2)	(3)	(4)	(5)	(6)	(7)	(8)
Constant	0.507 (0.453)	-0.017 (0.554)	4.603** (1.826)	2.220 (1.38)	4.799 (3.254)	1.613 (2.919)	7.467 (3.376)**	5.208 (3.274)
log(*ihc*)	0.242*** (0.065)		0.552*** (0.138)		0.580*** (0.147)		0.430* (0.229)	
log(*AYE*)		1.082*** (0.277)		1.875*** (0.439)		1.586*** (0.551)		1.042** (0.498)
log(GDP75)			-0.697** (0.293)	-0.441** (0.211)	-0.954*** (0.291)	-0.762*** (0.239)	-0.953*** (0.321)	-0.919*** (0.230)
N	91	91	91	91	91	91	91	91
*R*^2^	0.189	0.169	0.343	0.223	0.429	0.345	0.458	0.452
Hansen J statistic (p-value)	0.625	0.074	0.809	0.045	0.947	0.097	0.248	0.092

Note: Huber-White standard errors. All variables are averages for the period 1975-2011. External instruments are initial GDP in 1975 and its lags in the period 1970. Statistical significance:

*p<0.1,

**p<0.05,

***p<0.01.

Where: *ihc*, the index of human capital; *AYE*, average years of education; GDP75, initial value of GDP. Controls used: government participation as a percentage of GDP, inflation, investment in physical capital to GDP, population growth rate, level of democracy, contestation, inclusiveness and a dummy for African countries. Although not reported, the validity of the instruments is tested, besides Hansen J statistic, by means of critical values Stock and Yogo (2003) from Kleibergen-Paap rk Wald F statistic which is robust to heteroskedasticity.

The coefficients of *ihc* remain highly significant to the first three specifications, and their impact ranges from 0.506 to 1.212. However, in the specification where all variables are included (Column 7), the statistical significance and the impact of *ihc* decreases. This result reflects the exclusion restrictions that we used. Although the Hansen J statistic shows that this model complies with the orthogonality conditions, an inspection by the Kleibergen-Paap rk Wald F statistic reveals its low value and points out some persistence in the weakness of the instruments used. We observed the same results in *AYE*, where the Hansen J statistic is not passed satisfactorily for any of the specifications. In order to find more consistent results, we now estimate the models using Lewbel’s approach. Table 3 shows these results.

**Table 3:** Regression analysis for economic growth-heteroskedasticity-based instruments.

	Dependent variable: log difference GDP							
	(1)	(2)	(3)	(4)	(5)	(6)	(7)	(8)
Constant	0.507 (0.453)	-0.017 (0.554)	3.603 (1.742)*	2.166 (1.377)	5.528* (2.881)	0.484 (2.833)	9.313*** (1.980)	4.893*** (1.798)
log(*ihc*)	0.242*** (0.065)		0.482*** (0.132)		0.628*** (0.137)		0.626*** (0.161)	
log(*AYE*)		1.082*** (0.277)		1.849*** (0.436)		1.364*** (0.507)		0.951** (0.389)
log(GDP75)			-0.535* (0.279)	-0.430** (0.210)	-1.237*** (0.242)	-0.804*** (0.225)	-1.255*** (0.234)	-0.829*** (0.163)
N	91	91	91	91	91	91	91	91
*R*^2^	0.189	0.169	0.320	0.223	0.353	0.289	0.449	0.435
Hansen J statistic (p-value)	0.625	0.074	0.159	0.122	0.281	0.151	0.387	0.115

Note: Huber-White standard errors. All variables are averages for the period 1975-2011. External instruments are initial GDP in 1975 and its lags in the period 1970. Statistical significance:

*p<0.1,

**p<0.05,

***p<0.01.

Where: *ihc*, the index of human capital; *AYE*, average years of education; GDP75, initial value of GDP. Controls used: government participation as a percentage of GDP, inflation, investment in physical capital to GDP, population growth rate, level of democracy, contestation, inclusiveness and a dummy for African countries. Although not reported, the validity of the instruments is tested, besides Hansen J statistic, by means of critical values Stock and Yogo (2003) from Kleibergen-Paap rk Wald F statistic which is robust to heteroskedasticity.

*ihc* preserves its statistical significance and passes endogeinity tests in all specifications. *AYE*, in contrast, did not pass the endogeinity test in the second specification (Column 2), and its explained variance always proved to be lower than the explained variance of *ihc*.

**Fig 2:**
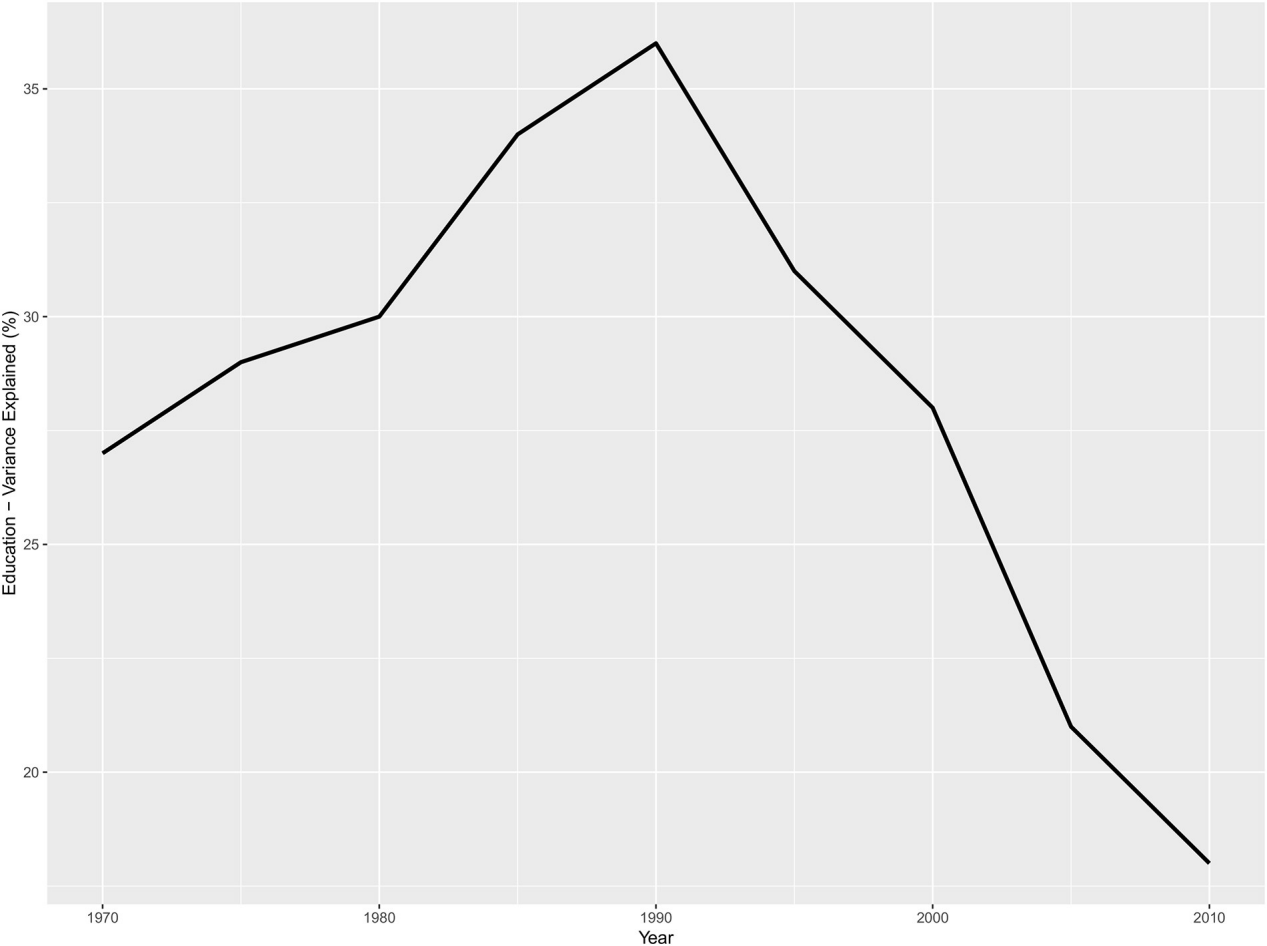
Contribution of the education block to the explanation of human capital. Parameters are estimated with PLS-PM as depicted in Fig 1 plus environment and resources as additional regressors.

On the other hand, an important fact that we have found in the construction of our *ihc* indicator, is that if we decompose the explained variance of each latent variable we observed that the predictive power of the set of educational variables increased from 1970 to 1990 but dwindled after then (see Fig 2).

**Fig 3:**
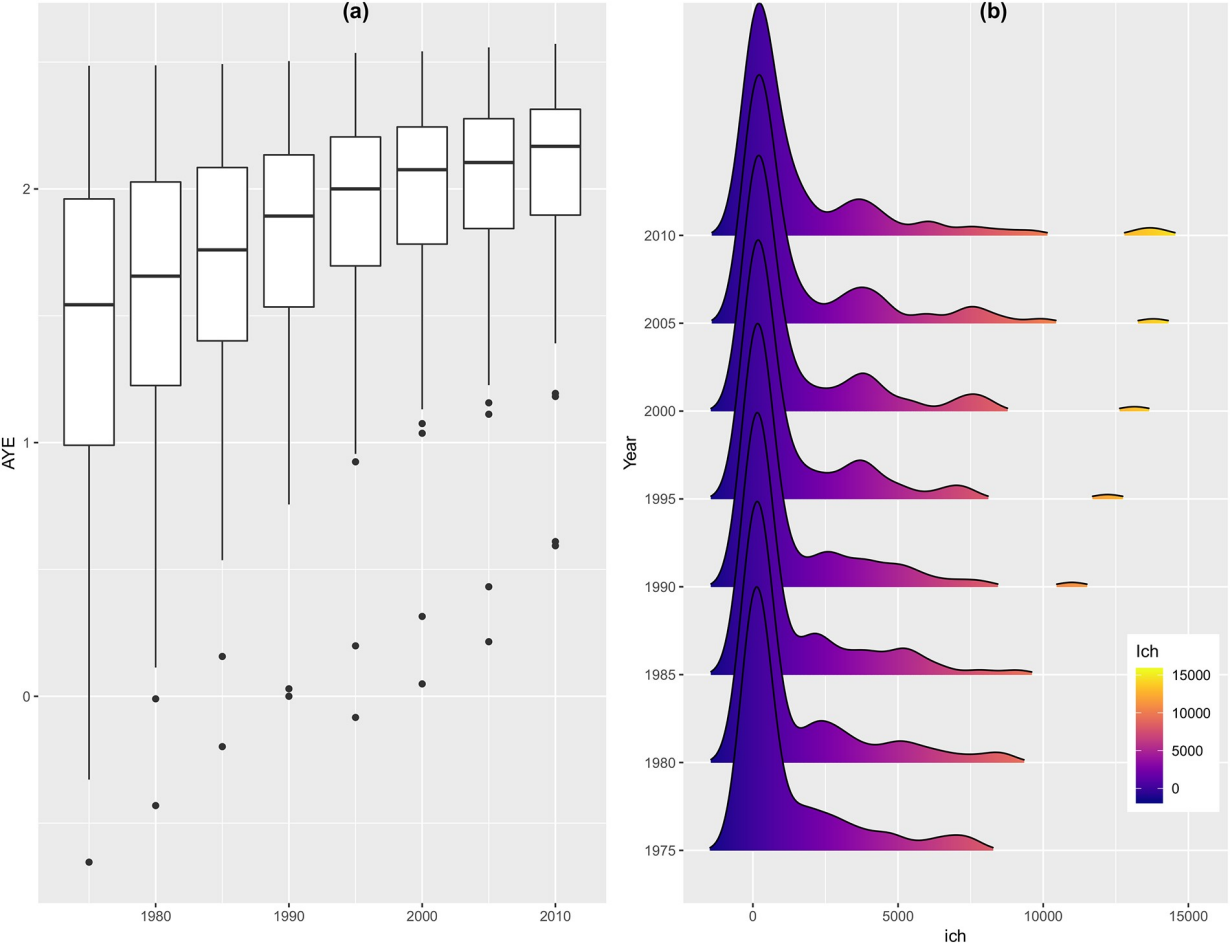
Performance of human capital variables over time. (a) Boxplot diagrams for Average Years of Education (*AYE*) as a function of time. (b) Statistical distribution of Human Capital Index (*ihc*) as a function of time.

Another interesting fact emerges when we compare nations schooling and our index of human capital. It turns out that the average years of education not only are systematically increasing worldwide but its variance is decreasing as time passes; a phenomenon that we can call “educational homogenization”. Our index, however, remains almost invariant throughout the time (see Fig 3).

**Fig 4:**
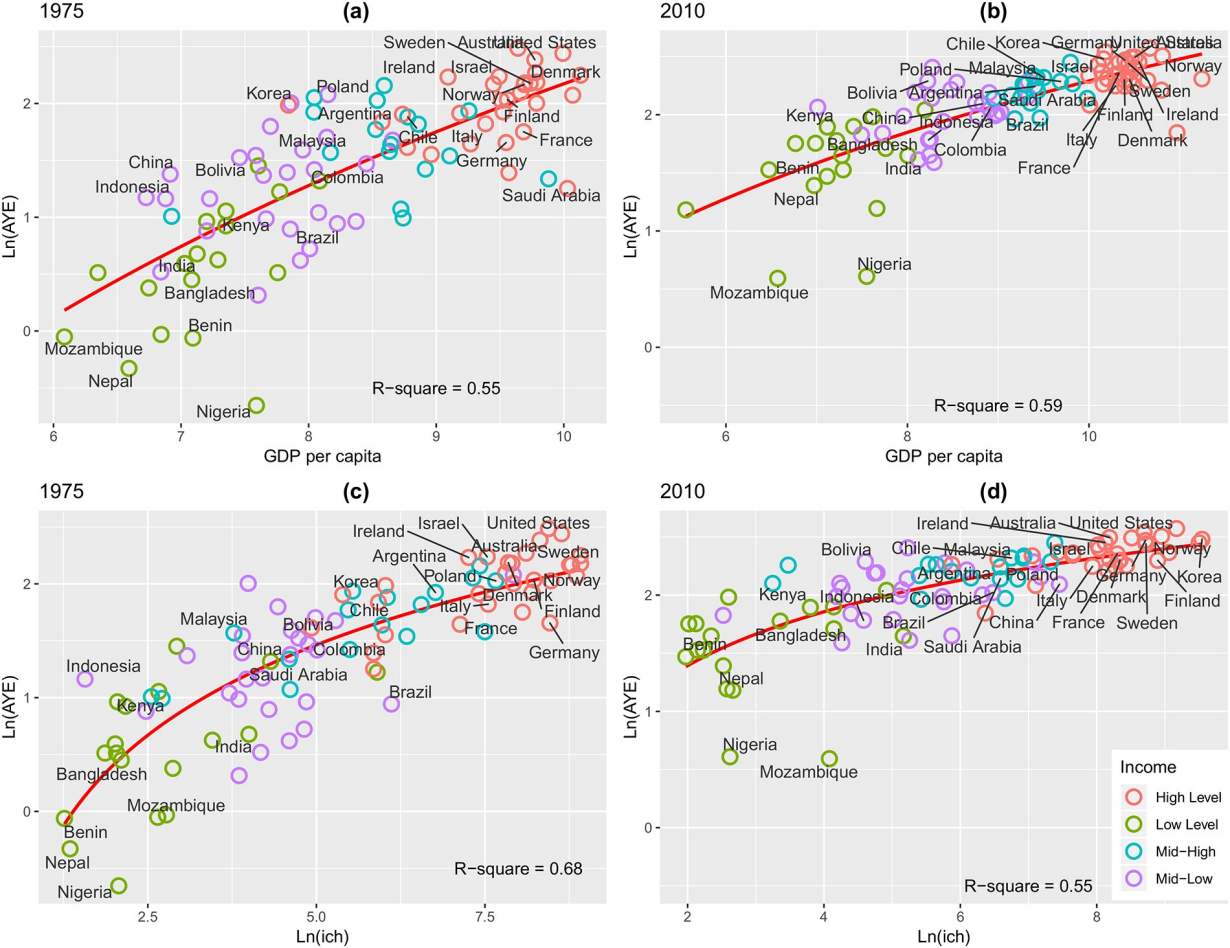
Correlation analysis between different variables of human capital and well-being. (a) Scattergram for GDP per capita and *AYE* in 1975. (b) Scattergram for GDP per capita and *AYE* in 2010. (c) Scattergram for *ihc* and *AYE* in 1975. (d) Scattergram for *ihc* and *AYE* in 2010.

When the average years of education is regressed on economic wealth, as captured by GDP per capita, we noticed that this association seems to increase slightly, given the *R*^2^ difference between 1975 and 2010. However, the relationship between wealth and education reveals a different story. Once again, schooling of countries is becoming homogeneous while human capital remains heterogeneous throughout time and across countries. Fig 4 and Table 4 summarize these results.

**Table 4:** Regression analysis for economic growth by periods.

	Dependent variable: log difference GDP					
	Period, 1975-1990					
	OLS	OLS	GMM	GMM	Lewbel (2012)	Lewbel (2012)
ln(*ihc*)	1.037*** (0.342)		0.655 (0.442)		0.925*** (0.279)	
ln(AYE)		3.313*** (0.930)		2.236** (1.119)		3.108*** (0.714)
N	91	91	91	91	91	91
*R*^2^	0.447	0.477				
Centered *R*^2^			0.425	0.458	0.410	0.401
Hansen J statistic (p value)			0.0539	0.0764	0.3378	0.4772
	Period, 1991-2011					
	OLS	OLS	GMM	GMM	Lewbel (2012)	Lewbel (2012)
ln(ihc)	0.856** (0.362)		0.448 (0.329)		0.446** (0.199)	
ln(AYE)		-1.397 (1.429)		-1.518 (1.228)		-0.138 (0.758)
N	91	91	91	91	91	91
*R*^2^	0.279	0.187				
Centered *R*^2^			0.127	0.077	0.244	0.149
Hansen J statistic (p value)			0.8818	0.9084	0.3777	0.4178

Note: All variables are averages for the underlying period. External instruments are initial GDP in 1975 and its lags in the period 1970. Statistical significance:

*p<0.1,

**p<0.05,

***p<0.01.

Although not reported, the validity of the instruments is tested, aside from the Hansen J statistic, by means of critical values Stock and Yogo (2003) from the Kleibergen-Paap rk Wald F statistic which is robust to heteroskedasticity.

We confirmed the importance of *AYE* in determining economic growth between 1975 and 1990. Considering the different estimation methods, *AYE* shows a strong and significant impact, although again, full identification is only achieved through Lewbel’s approach. Meanwhile, *ihc* shows its good performance as a proxy of human capital, although in the case of IV by GMM, this variable is not significant due to the invalidity of the instruments (i.e., it does not pass the Hansen J statistic). Once the model is estimated by heteroskedasticity-based instruments, the coefficient of *ihc* shows a strong impact and is highly significant, while successfully correcting the problems of endogeneity.

The stagnation of *AYE* and the homogenization between countries is impacting significantly on its power of explication on economic growth. In each of the specifications, this variable not only is no longer statistically significant but also presents the wrong sign. Although *ihc* loses statistical significance, it continues to show good performance, particularly when endogeneity problems are corrected. These results show that *AYE* is losing explanatory capacity as time passes, due to its limitations and the thresholds reached by this variable. It is evident that when human capital incorporates other variables that go beyond a single educational metric based on quantity, its explanatory power improves substantially.

To assess the behavior of these indicators in relation to the level of development we show the results when we exclude the countries that belong to the OECD in Table 5. Both indicators behave similarly. Although *AYE* proved to be a significant predictor, such a relationship is misleading because its statistical significance disappears when evaluated with panel data.

**Table 5:** Regression analysis for economic growth by sample of countries.

	Dependent variable: log difference GDP					
	OLS	OLS	GMM	GMM	Lewbel (2012)	Lewbel (2012)
ln(ihc)	0.667*** (0.226)		0.630** (0.272)		0.506*** (0.122)	
ln(AYE)		1.221*** (0.479)		1.104** (0.54)		0.938*** (0.352)
Sample size	60	60	60	60	60	60
*R*^2^	0.4894	0.438				
Centered *R*^2^			0.4254	0.418	0.4483	0.3949
Hansen J statistic (p value)			0.5165	0.2398	0.4954	0.3896

Note: All variables are averages for the underlying period. External instruments are initial GDP in 1975 and its lags in the period 1970. Statistical significance:

*p<0.1,

**p<0.05,

***p<0.01.

Although not reported, the validity of the instruments is tested, aside from the Hansen J statistic, by means of critical values Stock and Yogo (2003) from the Kleibergen-Paap rk Wald F statistic which is robust to heteroskedasticity.

The results of this specification are shown in Table 6 by means of a balanced panel and data averaged every five years during the period 1975-2011. The table divides the estimates by placing the models of the index of human capital at the top, and the educational variable (average years of education) at the bottom. Following Bond et al. (2001) regressions by pooled OLS and fixed-effects (FE) are performed. Both estimates use robust standard errors, clustered by country. These estimates are informative because they give the lower and upper limits for the autoregressive coefficient of GDP.

**Table 6:** Estimation by the system GMM estimator (full sample).

	Pooled OLS	OLS FE	SYS- GMM	Pooled OLS	OLS FE	SYS- GMM
	(1)	(2)	(3)	(4)	(5)	(6)
	I. Regressions to ihc, dependent variable log difference GDP					
Log(lagged GDP)	-0.201*** (0.043)	-0.686*** (0.065)	-0.619*** (0.107)	-0.201*** (0.048)	-0.688*** (0.068)	-0.472*** (0.114)
Log(*ihc*)	0.121*** (0.019)	0.066 (0.05)	0.304*** (0.065)	0.069*** (0.019)	0.063 (0.047)	0.138* (0.08)
Controls	Not	Not	Not	Yes	Yes	Yes
Instruments			26			50
Hansen J statistic			[0.630]			[0.278]
AR(1)			[0.000]			[0.000]
AR(2)			[0.443]			[0.216]
Observations	616	616	616	616	616	616
Countries	91	91	91	91	91	91
*R*^2^	0.161			0.209		
*R*^2^ within		0.313			0.343	
	II. Regressions to AYE, dependent variable log difference GDP					
Log(lagged GDP)	-0.128*** (0.036)	-0.683*** (0.067)	-0.379*** (0.089)	-0.169*** (0.041)	-0.688*** (0.069)	-0.347*** (0.078)
Log(*AYE*)	0.360*** (0.071)	-0.239 (0.186)	1.154*** (0.177)	0.196*** (0.054)	-0.304 (0.194)	0.681*** (0.206)
Controls	Not	Not	Not	Yes	Yes	Yes
Instruments			26			50
Hansen J statistic			[0.287]			[0.673]
AR(1)			[0.000]			[0.001]
AR(2)			[0.282]			[0.163]
Observations	616	616	616	616	616	616
Countries	91	91	91	91	91	91
*R*^2^	0.115			0.203		
*R*^2^ within		0.311			0.344	

Note: Statistical significance:

*p<0.1,

**p<0.05,

***p<0.01.

The sample is a balanced panel covering the period 1975-2011. In pooled OLS and fixed effects regression models the errors are robust. In the System GMM estimator is estimated using two-step and uses the Windmeijer’s correction for errors. In regressions (4) to (6) used as controls: logarithms of investment, population growth, consumption as a percentage of GDP, inflation, institutional variables, and a dummy for African countries. The lagged GDP is treated as a predetermined variable, while *ihc*, *AYE*, investment and population growth as endogenous.

The effect of human capital on economic growth is first observed without incorporating control variables, except time dummies, columns (1) to (3). The lagged GDP is treated as a predetermined variable, while *ihc* and *AYE* are treated as endogenous variables. As seen, the pooled OLS estimates (column 1) and fixed-effects (column 2) show that the limits for the lagged GDP, are located in the range of [-0.686, -0.201] for those models that include *ihc* and [-0.683, -0.128] wherein *AYE* is present; in both cases the coefficients are negative and highly significant.

Both *ihc* and *AYE* have coefficients with positive signs and both are highly significant in pooled OLS models, but *ihc* shows a greater explanatory power. On the other hand, those variables in the estimates by fixed-effects do not appear to contribute much to the model; both are not significant and *AYE* shows a negative sign, which may be caused by potential bias from endogeneity problems and the lagged GDP value.

In columns (4) and (5) these same models are estimated by introducing the set of controls presented in the section of data. As noted, although the coefficients of *ihc* and *AYE* decreased, in the light of these new specifications, both indicators have similar performance to previous models. Column (3) shows the results for the system GMM estimator (two-step), employing only time dummies and the second lag to avoid the proliferation of instruments. We used Windmeijer’s correction for standard errors [41]. For both models of human capital, those with *ihc* and *AYE*, the lagged GDP is within the limits set by columns (1) and (2), and has the expected sign with a high level of significance. Both proxies of human capital show positive coefficients which are highly significant. An increase of one standard deviation (1.7) in the proposed indicator increases GDP growth in 0.504, meanwhile, the educational variable does so at 0.642. On the other hand, when control variables are included in the regression models, *ihc* loses statistical significance, in opposition to *AYE* which remains highly significant for predicting economic growth.

The Hansen J statistic shows p-values for the null hypothesis of the validity of the over-identifying restrictions. None of the specifications reject the null hypothesis and thus indicate the validity of the instruments. AR(1) and AR(2) are the p-values for auto-correlated errors of the first and second order respectively, in the first differences equation. While AR(1) is expected to be significant, AR (2) is a specification test under the null hypothesis of no serial correlation. Again, none of the specifications can reject this hypothesis. These tests indicate an appropriate specification of the models.

As before and once both variables are validated by dynamic panels, we evaluated the two concerns raised in relation to the performance of *AYE* and the response of *ihc* to these specifications. First, Table 7 shows the results for both indicators, when only taking into account the period 1975-1990 and the same specifications as in Table 6. In this case, both *AYE* and *ihc* seem to perform well when not all the controls are used. When these are included both variables are no longer significant, they significantly reduce their impact, and fail in the identification of the models. The significant reduction of the instruments used in the identification may be influencing the results, because as shown by the pooled OLS estimation, both variables are highly significant, confirming the previous results.

**Table 7:** Sensitivity analysis (different periods, 1975-1990).

	Pooled OLS	OLS FE	SYS- GMM	Pooled OLS	OLS FE	SYS- GMM
	(1)	(2)	(3)	(4)	(5)	(6)
	I. Regressions to ihc, dependent variable log difference GDP					
Log(lagged GDP)	-0.268*** (0.045)	-0.271 (0.173)	-0.521*** (0.245)	-0.288*** (0.03)	-0.236 (0.212)	-0.292 (0.263)
Log(ihc)	0.147*** (0.02)	-0.161* (0.078)	0.537** (0.247)	0.084*** (0.02)	-0.173* (0.091)	0.031 (0.138)
Controls	Not	Not	Not	Yes	Yes	Yes
Instruments			6			20
Hansen J statistic			[0.186]			[0.047]
AR(1)			[0.014]			[0.010]
AR(2)			[0.000]			[0.000]
Observations	255	255	255	255	255	255
Countries	91	91	91	91	91	91
*R*^2^	0.288			0.3736		
*R*^2^ within		0.143			0.1776	
	II. Regressions to AYE, dependent variable log difference GDP					
Log(lagged GDP)	-0.216*** (0.042)	-0.300* (0.174)	-0.647*** (0.111)	-0.269*** (0.046)	-0.265 (0.207)	-0.463* (0.263)
Log(AYE)	0.496*** (0.086)	-0.594 (0.386)	1.584*** (0.211)	0.310*** (0.085)	-0.435 (0.307)	0.758 (0.727)
Controls	Not	Not	Not	Yes	Yes	Yes
Instruments			6			20
Hansen J statistic			[0.724]			[0.028]
AR(1)			[0.093]			[0.057]
AR(2)			[0.000]			[0.000]
Observations	255	255	255	255	255	255
Countries	91	91	91	91	91	91
*R*^2^	0.277			0.3916		
*R*^2^ within		0.14			0.1646	

Note: Statistical significance:

*p<0.1,

**p<0.05,

***p<0.01.

In pooled OLS and fixed effects regression models the errors are robust. In the System GMM estimator is estimated using two-step and uses the Windmeijer’s correction for errors [41]. In regressions (4) to (6) used as controls logarithms of investment, population growth, consumption as a percentage of GDP, inflation, institutional variables, dummy for African countries and time dummies. The lagged GDP is treated as a predetermined variable, while ihc, AYE, investment and population growth are treated as endogenous.

Hence, although restricting the sample to this sub-period does not allow to correct endogeneity problems satisfactorily, the estimates by pooled OLS allow to elucidate that *AYE* plays a crucial role in the formation of human capital, impacting on economic growth significantly. It is in this period that the highest growth rates in terms of quantity-based education occur worldwide, particularly in the seventies, with increasing levels of human capital and productivity, as reflected in the proposed indicator.

However, when the period under consideration is 1991-2011, *AYE* loses all relevance as a determinant of economic growth, even if no controls are included (Table 8).

**Table 8:** Sensitivity analysis (different periods, 1991-2011).

	Pooled OLS	OLS FE	SYS- GMM	Pooled OLS	OLS FE	SYS- GMM
	(1)	(2)	(3)	(4)	(5)	(6)
	I. Regressions to ihc, dependent variable log difference GDP					
Log(lagged GDP)	-0.140*** (0.048)	-1.479*** (0.277)	-1.04*** (0.287)	-0.095*** (0.059)	-1.562*** (0.268)	-0.493* (0.290)
Log(ihc)	0.075*** (0.027)	0.248*** (0.082)	0.566*** (0.174)	0.065* (0.033)	0.254*** (0.082)	0.333 (0.201)
Controls	Not	Not	Not	Yes	Yes	Yes
Instruments			8			18
Hansen J statistic			[0.333]			[0.351]
AR(1)			[0.047]			[0.026]
AR(2)			[0.000]			[0.000]
Observations	271	271	271	271	271	271
Countries	91	91	91	91	91	91
*R*^2^	0.0713			0.1236		
*R*^2^ within		0.3482			0.4413	
	II. Regressions to AYE, dependent variable log difference GDP					
Log(lagged GDP)	-0.017	-1.397*** (0.029)	-0.240* (0.272)	-0.001 (0.132)	-1.472*** (0.039)	-0.092 (0.174)
Log(AYE)	-0.046 (0.104)	0.294 (0.337)	1.049* (0.558)	-0.137 (0.109)	0.156 (0.332)	0.412 (0.510)
Controls	Not	Not	Not	Yes	Yes	Yes
Instruments			8			18
Hansen J statistic			[0.005]			[0.155]
AR(1)			[0.031]			[0.015]
AR(2)			[0.000]			[0.000]
Observations	271	271	271	271	271	271
Countries	91	91	91	91	91	91
*R*^2^	0.0208			0.1071		
*R*^2^ within		0.326			0.4185	

Note: Statistical significance:

*p<0.1,

**p<0.05,

***p<0.01.

In pooled OLS and fixed effects regression models the errors are robust. In the System GMM estimator is estimated using two-step and uses the Windmeijer’s correction for errors [41]. In regressions (4) to (6) used as controls logarithms of investment, population growth, consumption as a percentage of GDP, inflation, institutional variables, dummy for African countries and time dummies. The lagged GDP is treated as a predetermined variable, while ihc, AYE, investment and population growth are treated as endogenous.

In this case, *AYE* shows the wrong sign when the model is estimated by pooled OLS. Meanwhile *ihc* exhibits a better behavior when controls are not included (i.e., it shows a strong impact and it is highly significant in all specifications). With controls *ihc* loses significance when it is estimated by the system GMM estimator, which again can be explained by the small number of instruments, leading to identification failure. However, using other methods, *ihc* proved to be significant. The performance of *ihc* can also be explained by the inner dynamics of its indicators, including AYE. The marginal increases in educational variables based on quantity do not seem to be enough to influence the performance of human capital and, thus, productivity and economic growth. In this most recent period, factors related to the quality of human capital accumulation seem to be more influential on the performance of these variables. Given this scenario, *ihc* performs better, and this is because within its structure there are variables that somehow better approximate the quality of human capital. This indicator, however, shows limitations, mainly as a consequence of the availability of data to better measure other elements of the quality of this stock and, secondly, because the variables used in its development lead the indicator to stationary points.

Another concern proposed above was related to the performance of *ihc* and *AYE* when tackling differences in countries levels of development. Table 9 shows this analysis by splitting the sample in two sub-sets of data: first, columns (1) and (2) show the system GMM estimator estimates excluding to the high-income countries and Asian tigers (Korea and Singapore). Second, in columns (3) and (4) we only included non-OECD countries. As noted, while the proposed indicator *ihc* always shows the correct sign, with a statistically significant impact, AYE is not significant to the inclusion of all control variables. Furthermore, the test specification of *ihc* performs well in all regressions, while for *AYE*, it does not pass the Hansen J statistic when no controls are included.

**Table 9:** Sensitivity analysis (different samples).

	SYS-GMM (Without Rich and Asian Tigers)	SYS-GMM (Without Rich and Asian Tigers)	SYS-MGM (Excluding OECD)	SYS-MGM (Excluding OECD)
	(1)	(2)	(3)	(4)
	I. Regressions to ihc, dependent variable log difference GDP			
Log(lagged GDP)	-0.443** (0.188)	-0.463** (0.188)	-0.654*** (0.141)	-0.490*** (0.112)
Log(ihc)	0.203** (0.099)	0.209*** (0.071)	0.315*** (0.087)	0.231** (0.108)
Controls	Not	Yes	Not	Yes
Instruments	26	50	26	50
Hansen J statistic	[0.622]	[0.258]	[0.586]	[0.346]
AR(1)	[0.002]	[0.005]	[0.001]	[0.002]
AR(2)	[0.458]	[0.263]	[0.550]	[0.272]
Observations	415	415	408	408
Countries	62	62	61	61
	II. Regressions to AYE, dependent variable log difference GDP			
Log(lagged GDP)	-0.332* (0.171)	-0.174 (0.159)	-0.399*** (0.101)	-0.235** (0.095)
Log(AYE)	0.828*** (0.222)	0.205 (0.296)	0.865*** (0.251)	0.346 (0.287)
Controls	Not	Yes	Not	Yes
Instruments	26	50	26	50
Hansen J statistic	[0.096]	[0.348]	[0.078]	[0.241]
AR(1)	[0.004]	[0.009]	[0.001]	[0.003]
AR(2)	[0.428]	[0.191]	[0.382]	[0.172]
Observations	415	415	408	408
Countries	62	62	61	61

Note: Statistical significance:

*p<0.1,

**p<0.05,

***p<0.01.

In pooled OLS and fixed effects regression models the errors are robust. In the System GMM estimator is estimated using two-step and uses the Windmeijer’s correction for errors [41]. In regressions (4) to (6) used as controls logarithms of investment, population growth, consumption as a percentage of GDP, inflation, institutional variables, dummy for African countries and time ummies. The lagged GDP is treated as a predetermined variable, while ihc, AYE, investment and population growth are treated as endogenous.

As explained above, the facts show that *AYE* not only is reducing its growth rates, but it is also homogenizing around a level which possibly is difficult to escape by the cost-benefit ratio that implies increases in this investment. These trends together with the limitations of this indicator are likely to reduce the ability to influence on effective increments in human capital and, hence, on economic growth. The indicator *ihc* shows a superior performance in this context because it captures better the disparities in terms of human capital between countries. The differences in the scores of the proposed indicator better characterize the differences regarding productivity and the quality of education between countries.

## Discussion

The aim of this paper was to propose a new index of human capital whose direct and indirect effects could be estimated so as to disentangle the relationships that exist between the process of accumulating knowledge required to produce economic value (e.g., education, health, household background) and their returns and outcomes (e.g., productivity, generation of new knowledge, etc.).

By being based on a structural equation modeling approach, our index allows an ample definition from the viewpoint of latent variables in social sciences [14]. Such an approach not only shows the multidimensional nature of human capital, but it also enables further modifications by excluding some of the observable variables that we used or even including new ones not considered in this first exploration.

Given the availability of data, we were able to report *ihc* for a reasonably extended period of time ranging from 1970 to 2011. Besides, we were able to extend the results shown by Messinis and Ahmed [12], by comparing the robustness of this new latent variable with the average years of education in both cross-section data and panel data, and test the hypothesis that the positive effect of schooling could only be observable after nations have crossed an educational threshold [16].

We have shown that our index performed better than existing measures as it tackles omitted variable bias, and prevents the limitations of information reduction techniques such as principal component analysis. The proposed measure showed good performance concerning different specifications and econometric techniques to explain economic growth. The proposed indicator overcame some weaknesses with a warning. Rather than including *AYE* in the block of educational variables, future research could focus on the International Cognitive Assessment (ICA) scores [6]. A precaution exists in this regard. The majority of available databases lack sufficient information for both the sample of countries and years. Altinok and Murseli [42], for example, built a cognitive indicator with a sample of various international tests. The problem with their indicator is that data are only consistently available for a small number of developing countries in the years 2000, 2003, 2005, 2007 and 2009. As this information is not entirely available for all nations during our observed period, it is essential to find and share historical records of these metrics because they might enhance the performance of our indicator.

Other improvements are indeed welcome in future efforts. For example, rather than including the number of patents per capita as a proxy of innovation, future research could include scientific productivity in physical and chemical science since they predict the future economic growth of countries better than other popular indices [43]. Corruption perception index might be also integrated as another proxy of socio-economic conditions of countries [44, 45]. Searching for better available variables might help us refine the scientific endeavor of a better approach to human capital.
